# Neurosurgery and Spine Surgery: From Up-to-Date Practitioners, From the Past to the Present

**DOI:** 10.3390/jcm13195840

**Published:** 2024-09-30

**Authors:** Massimiliano Visocchi

**Affiliations:** 1 Institute of Neurosurgery Catholic Fondazione Policlinico Agostino Gemelli, Catholic University Rome, 00153 Rome, Italy; mvisocchi@hotmail.com; 2 Craniovertebral Junction Surgery Operative Unit, Research Center Catholic University, 00168 Rome, Italy

The Special Issue titled “Neurosurgery and Spine Surgery: From Up-to-Date Prac-titioners” provides a comprehensive overview of the latest advancements and best practices in the fields of neurosurgery and spine surgery. This collection of articles showcases cutting-edge research, techniques, and clinical experiences from leading ex-perts in these disciplines. The Special Issue focuses on research articles and reviews, emphasizing the exclusion of case reports to maintain a high level of scientific rigor. Topics covered include innovative surgical approaches, emerging technologies, periop-erative care, patient outcomes, and interdisciplinary collaboration in the treatment of neurological and spinal disorders. This compilation serves as a valuable resource for healthcare professionals, researchers, and students interested in staying current with the rapidly evolving landscape of neurosurgery and spine surgery. The contributions of seasoned practitioners shed light on the most pressing issues and promising develop-ments in these crucial medical specialties. Chiari malformation; syringomyelia; foramen magnum decompression.

According to a recent theory, the skull base and spine complex morphologically reproduces a funnel.

“A funnel is an object that has a wide round opening at the top, sloping sides, and a narrow tube at the bottom, used for pouring liquids or powders into containers with narrow necks” according to the Cambridge Dictionary, [1].

A real bone funnel seems to be replicated by the skull base along with its offshoot, the spine; it resembles a vessel containing the brain, the cerebellum, and the spinal cord together with cranial and radicular nerves. In my opinion, the knowledge of embryology, anatomy, physiology, pathophysiology, and the most effective surgical options to effectively surgically remove diseases is mandatory for building a real neurosurgical library to be suggested to young neurosurgeons. The “Neurosurgery and Spine Surgery: From Up-to-Date Practitioners, 2nd Edition” Special Issue provides a comprehensive summary of the latest advancements and best practices in the neurosurgery and spine surgery fields, including the skull base and its content. This collection of articles shows cases, cutting-edge research, techniques, and clinical experiences from leading experts in these disciplines. The Special Issue focuses on research articles and reviews, emphasizing the exclusion of case reports to maintain a high level of scientific rigor. The topics covered include innovative surgical approaches, emerging technologies, perioperative care, patient outcomes, and interdisciplinary collaboration in the treatment of neurological and spinal disorders. The aim of this Special Issue is to allow a natural evolution of classic neurosurgery from a totally demolitive philosophy to a new concept of “restorative demolition”. In this New Deal of neurosurgical philosophy, new concepts such as reconstruction (historically the term “reconstruction” was intended in a purely mechanical but not “functional” way), restoration, or rehabilitation should not be confused with a simple old-fashioned demolition plan, but need to be merged with the new heritage of high-tech neurosurgical repair procedures [2]. Moreover, we focused our attention on new tools in neurosurgical practice: endoscopy, exoscopy, augmented reality, minimally invasive procedures, and neuromonitoring, and highlighted the interest in complex spine surgery as craniocervical junction (CVJ) neurosurgery (Figure 1 and Figure 2) [3,4,5]. Strong consideration was recently gained by CVJ surgery or the CVJ specialty in neurosurgery, as a top-level, challenging, exclusive, and selective form of surgery, which is also a paradigm of a true frontier surgery. Managing lesions situated in the anterior aspect of the CVJ remains a neurosurgical challenge, although the beginnings of this pioneering surgery precede this century. The CVJ not only separates the skull base from the subaxial cervical spine but also allows a unique cranial flexion, extension, and axial rotation pattern due to its unique anatomical neurovascular and bone ligamentous architecture. To better understand the occiput, atlas, and axis instrumentation procedures as well as the specific diseases affecting this unique region, modern neurosurgeons need to deepen their knowledge of not only the basic principles of surgical instrumentation but also, if not mostly, the local kinematics. In fact, perfect knowledge of CVJ anatomy (historically considered a no man’s land) and movement physiology is required for correct and effective preoperative planning and to obtain a brilliant postoperative result [6]. Neuromodulation is also a challenging neurosurgical issue that is progressively gaining widespread application. Classically, neuromodulation is considered a special surgical therapy for chronic pain and spasticity; nevertheless, many other surgical options exist to deal with the treatment of Parkinson’s disease, such as deep brain stimulation (DBS), but also spinal and sacral nerve stimulation for bowel and bladder dysfunction and generically pelvic disorders. Surprisingly, spinal cord stimulation has also been indicated for ischemic disorders (angina, peripheral vascular disease) and vegetative states. Personally, I have contributed in the past to a better understanding ischemic mechanisms and related therapeutic options with spinal cord stimulation [7,8,9].

Finally, brain tumor treatment is one of the most debated issues. A multidisciplinary approach is required to merge the best therapeutic experiences of different specialists like oncologists, radiotherapists, and surgeons. The extent of tumor resection is determinant in proceeding with a radical therapeutic strategy, and a multimodal intraoperative imaging protocol is also required. So far, a combination of neuromonitoring-guided resection, neuronavigation, intraoperative ultrasound (i-US), and intraoperative computed tomography (i-CT), along with 5-ALA fluorescence, seems to be promising in neurosurgical oncology [10,11,12].

The compilation of this Special Issue serves as a valuable resource for healthcare professionals, researchers, and students interested in staying current with the rapidly evolving landscape of neurosurgery and spine surgery. The contributions of seasoned practitioners shed light on the most pressing issues and promising developments in these crucial medical specialties. In conclusion, intelligence and culture, as well as knowledge of anatomy, pathology, and pathophysiology, seem to be strategic to facing a 360° universe of functions and diseases in neurosurgery.

Finally, the different skills of a multidisciplinary joining of worlds currently seem necessary to successfully deal with the final winning strategy; otherwise, technical skills and manual supports remain the personal heritage of the surgeon, absolutely necessary but not determinant of the postoperative result and long-term prognosis.

## Figures and Tables

**Figure 1:**
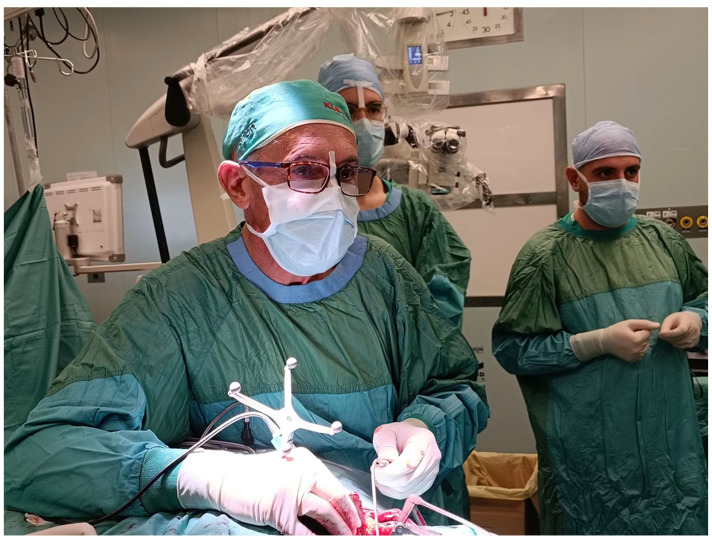
Neuronavigation in the surgical approach to the CVJ (personal experience).

**Figure 2:**
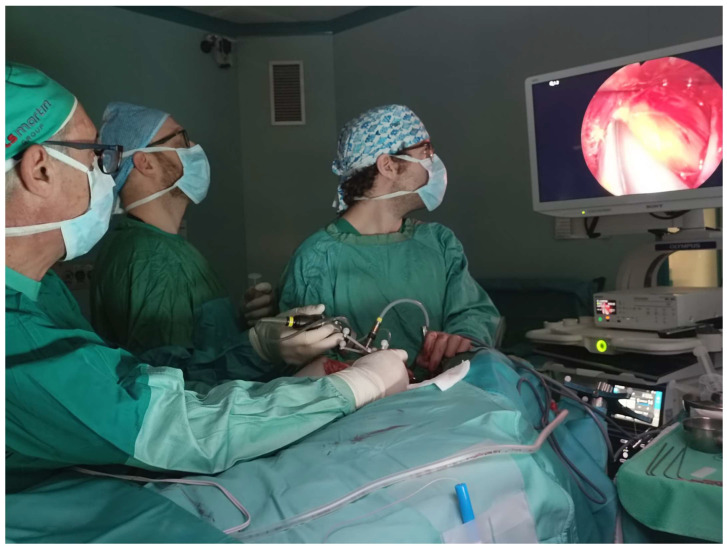
Endoscopy in the submandibular retropharingeal approach to the CVJ (personal experience).
